# Switching to second line MS disease-modifying therapies is associated with decreased relapse rate

**DOI:** 10.3389/fneur.2023.1243589

**Published:** 2023-09-06

**Authors:** James John Marriott, Okechukwu Ekuma, Randall Fransoo, Ruth Ann Marrie

**Affiliations:** ^1^Division of Neurology, Department of Medicine, St. Michael’s Hospital, University of Toronto, Toronto, ON, Canada; ^2^Manitoba Centre for Health Policy, Max Rady College of Medicine, Rady Faculty of Health Sciences, University of Manitoba, Winnipeg, MB, Canada; ^3^Department of Community Health Sciences, Max Rady College of Medicine, Rady Faculty of Health Sciences, University of Manitoba, Winnipeg, MB, Canada; ^4^Section of Neurology, Department of Medicine, Max Rady College of Medicine, Rady Faculty of Health Sciences, University of Manitoba, Winnipeg, MB, Canada

**Keywords:** multiple sclerosis, disease-modifying therapy, treatment efficacy, MS relapse, MS epidemiology

## Abstract

**Background and objectives:**

While randomized, controlled trials (RCTs) are the gold standard for determining treatment efficacy, they do not capture the effectiveness of treatment during real-world use. We aimed to evaluate the association between demographics and multiple sclerosis (MS) disease-modifying therapy (DMT) exposure, including treatment adherence and switches between different DMTs, on the risk of subsequent MS relapse.

**Methods:**

All persons with relapsing-onset MS (pwRMS) living in Manitoba between 1999 and 2014 were identified from provincial healthcare databases using a validated case definition. Use of DMTs was abstracted from the provincial drug database covering all residents of Manitoba, including use of any DMT, stopping/starting any DMT, switches between different DMTs and adherence as defined by cumulative medication possession ratios (CUMMPRs) of 50, 70, 80 and 90%. Time to first-treated relapse was used as the outcome of interest in logistic regression and Cox-proportional hazards regression models adjusting for demographic covariates including age and year of diagnosis, sex, socioeconomic status and number of medical comorbidities.

**Results:**

1780 pwRMS were identified, including 1,510 who were on DMT at some point in the study period. While total DMT exposure was not associated with the time to subsequent treated relapse, individuals who switched between more than 2 DMTs had higher post-switch rates of relapse. Switching to second-line DMTs was associated with a longer time to treated relapse in comparison to those who remained on a first-line DMT (HR 0.44; 95%CI: 0.32–0.62, *p* < 0.0001).

**Discussion:**

Switching to high-efficacy DMTs reduces the rates of subsequent MS relapse at the population level.

## Introduction

While randomized, controlled trials (RCTs) are the gold standard for determining treatment efficacy, they do not capture the effectiveness of treatment during real-world use. RCTs of disease-modifying therapies (DMTs) for relapsing multiple sclerosis (MS) are short (2–3 years) in a condition that spans decades and are conducted in a highly selected population typically with active disease, and excluding individuals with serious comorbidities (1). In routine clinical practice however, DMTs are prescribed for many years, often in individuals with less active MS, who may have comorbidities. RCTs comparing higher-potency DMTs are also lacking, as are RCTs comparing switching strategies after suboptimal treatment response. Real-world understanding of DMT effectiveness, including comparative effectiveness, is important for decision-making at the individual level by the person with MS and their clinicians. It is also important at the health system level. For example, a demonstration that higher-potency DMT improved long-term outcomes and costs related to relapses and disability progression might change policies that restrict DMT access.

Previous observational studies that have examined the effectiveness of DMT in real world settings have failed to account for treatment adherence (2), a particularly important concern when comparing different DMTs or switching strategies (3). This is important because therapies are ineffective when not used as prescribed, and adherence can vary according to therapy-specific factors (4). Therefore, it is important to account for the possibility that improved outcomes with change in therapy are not simply due to changes in adherence. To better capture the relationship between DMT exposure, including adherence and outcomes, we used a recently described model (5) that incorporates current DMT use (on/not on any DMT), cumulative DMT exposure at the time of the outcome and switches between different DMTs. We focused on severe relapses (defined as those requiring treatment) because they are an important patient-centered outcome which are more likely to lead to fixed disability (6, 7). We hypothesized that DMT use in general, and switches to higher-potency DMTs in particular, would be associated with lower rates of severe relapses.

## Methods

### Setting and data sources

This retrospective cohort study was conducted in Manitoba, Canada. Manitoba is centrally located in Canada with a population of approximately 1.4 million. Health care is universal and publicly funded for medically necessary services, including hospitalizations and physician services. The costs of some medications, such as disease-modifying therapies, may be partially or fully covered.

We used the administrative databases held in the Manitoba Population Research Data Repository at the Manitoba Centre for Health Policy (MCHP), including comprehensive health claims data for 98% of the provincial population; and the Winnipeg MS Clinic Registry (MSCR). The Population Registry database contains demographic information (age, sex, region of residence) and dates of health care coverage. The Discharge Abstract Database captures hospital admission and discharge dates, and discharge diagnoses using the International Classification of Disease (ICD) codes; [4-digit ICD 10th edition, Canadian enhancement (ICD-10-CA) codes since 2004 and 5-digit (ICD) 9th edition, clinical modification (ICD-9-CM) codes before 2004]. Physician claims data including dates and diagnoses using 3-digit ICD-9-CM codes were available in the Medical Services database. DMT utilization was derived from the Drug Program Information Network (DPIN) database, covering all prescriptions dispensed in the community regardless of payment source. The Winnipeg MS Clinic is the sole specialty clinic serving persons with MS (pwMS) in the province. In Manitoba, DMTs are only covered through the provincial Pharmacare program if they are prescribed through the MS clinic. The MSCR captures diagnosis and clinical course (clinically isolated syndrome, relapsing remitting, secondary progressive, primary progressive) which can be linked to the provincial databases using a scrambled, anonymized version of each resident’s personal health information number (PHIN).

### Protocol approvals and patient consents

The University of Manitoba Health Research Ethics Board approved this study. The Manitoba Health Information Privacy Committee approved administrative data access and linkage of the MSCR to the administrative data. Only MS clinic patients who consented to MSCR registry participation and data linkage were included in the data analysis; 89% of those approached have consented to participate. As required by privacy regulations, the precise values of all cell sizes <5 were suppressed for confidentiality reasons.

### Study population

All pwMS living in the province of Manitoba between fiscal years 1999/2000 (the year after formal establishment of the Manitoba MS Clinic) and 2014/2015 were identified by applying a validated case definition (8). This case definition requires at least 3 hospital, physician or prescription claims for MS ever, in any combination; it has a positive predictive value if 99.5% and negative predictive value of 97.5%. Hospital and physician claims for MS were identified using ICD-9-CM (340) or ICD-10-CA (G35) codes (ICD codes listed in Supplementary Table). MS-specific DMTs were identified using Drug Information Numbers (DINs). At the time this study was conducted the following DMTs were available through the provincial Pharmacare program: interferon-beta1b (Betaseron), interferon-beta1a (Rebif, Avonex), glatiramer acetate (Copaxone), natalizumab (Tysabri), fingolimod (Gilenya), Teriflunomide (Aubagio), and dimethyl fumarate (Tecfidera). Age at MS diagnosis was defined as the first year an International Classification of Disease (ICD)-9/10 claim code for any central nervous system demyelinating disease was recorded (8). As the health claims data do not include information on MS phenotype, we linked these data with the MSCR to limit the analysis to individuals with relapsing-onset pwMS (pwRMS) as previously described (8), since individuals with primary progressive MS were not eligible for DMTs.

### Exposure

Drug effectiveness can be a consequence of several factors including whether a drug is currently being used, cumulative exposure to that drug which effectively incorporates adherence, and number of changes in drug status (either on and off that medication) (5). This can be represented using three time-dependent variables and the interactions between them. First, DMT exposure was characterized as any DMT use (yes/no), beginning on the date of first dispensation, in any given year in the study period. The number of alterations in DMT use (shifts between yes/no status) was characterized as 0, 1 or ≥ 2; we did not distinguish shifts on and off the initial therapy versus changes between DMTs. Cumulative exposure was defined by cumulative medication possession ratios (CUMMPR). Medication possession ratios refer to the sum of the days’ supply of a particular drug in a specific time period, divided by the number of days in that period and multiplied by 100. When comparing individuals over the same period of time, the CUMMPR will be lower among individuals with lower adherence to treatment. Then all two- and three-way interaction terms between presence or absence of DMT exposure, number of alterations in DMT use and CUMMPR were incorporated.

We classified DMT switches as from any first line DMT (all interferon-beta and glatiramer products, dimethyl fumarate, teriflunomide as classified by the Manitoba Pharmacare program) to either: (i) another first line DMT, (ii) or to any second line therapy (fingolimod, natalizumab at the time of the study). In Manitoba, access to second-line therapy is limited to individuals who have relapses and MRI activity after at least 6 months on a first-line DMT or intolerance to two or more first-line agents.

### Outcome

The outcome of interest was time to first relapse requiring treatment, as these are clinically meaningful because they are associated with greater residual disability, as well as elevated health-care costs (6, 7). Moreover, disabling relapses are one of the criteria for access to second-line therapies in Manitoba. We have previously shown that such severe relapses can be reliably identified in Manitoba health databases using an algorithm including prescriptions for high dose oral corticosteroids (>500 mg/day for 3–11 days) or emergency room visits or hospitalizations for MS as most responsible diagnosis (positive predictive value [PPV] 100%, negative predictive value [NPV] 96%) (9).

### Covariates

We included several covariates in the analysis including sex, age at MS diagnosis, socioeconomic status, comorbidities, and year of diagnosis. Socioeconomic status (SES) was based on the average household income as derived from linking postal code of residence to Canadian Census Data (10). SES scores were initially split into quintiles and but then dichotomized into low (first to third quintiles) and high (fourth and five quintiles) SES groups in subsequent analyses to minimize comparisons with small numbers of patients. Comorbidity status was determined with a modified version of the Charlson Comorbidity Index (CCI) as previously described (11), and was categorized as 0, 1 or ≥ 2 based on the distribution in the study population. Year of diagnosis was categorized as 1999/00–2002/03, 2003/04–2008/09, and 2009/10–2014/15. The MS Clinic and Pharmacare program for DMTs were funded in 1999. Natalizumab, the first second-line therapy available in Manitoba was available through Pharmacare beginning in 2010.

### Analysis

We summarized characteristics of the study population using descriptive statistics including mean [standard deviation (SD)], and frequency (percent).

We conducted a series of regression analyses to understand the association between DMT use, including DMT switches and relapses. First, the time to first-treated relapse (endpoint) was initially modeled in all subjects using a Cox proportional hazards regression model. Time zero time was the date of MS diagnosis. We treated DMT exposure as a time-dependent covariate (DMT = 1 for each year on any DMT, DMT = 0 for each year not on DMT) to avoid immortal time bias, and account for changes in DMT exposure over time. This model adjusted for other covariates (age at diagnosis [continuous], sex [female as reference group], year of diagnosis, SES [low as reference group], and number of Charlson comorbidities [0 comorbidities as reference group]).

The impact of switching between DMTs on the time to first relapse was examined in a second analysis limited to the subset of the total cohort who *ever* started DMT. We adjusted for non-DMT related covariates as before. DMT use (yes/no), number of alterations in DMT use (shifts on/off DMT) and CUMMPR were all included as time-dependent covariates, along with three interaction terms (5). This approach allowed us to more accurately model the association between real-world medication use and the rate (hazard) of subsequent relapses (5). We report adjusted hazard ratios and 95% confidence intervals for the association between DMT exposure and time to treated relapse, according to number of alterations in drug use (on/off), for four CUMMPR thresholds of 50, 70, 80%, or 90%.

Finally, the association between switching from a first- to second-line DMT and time to first relapse post-switch was examined. This analysis included only those subjects who started DMT (with two subjects who exceptionally started on a second-line drug being excluded). In this model, the time on second line versus first-line DMT was treated as a time-dependent covariate. This model could not incorporate number of switches which was closely related to the type of DMT switch, thus we could not implement the same analysis as we did for “any DMT use” as described above.

All analyses were performed using SAS® version 9.4 (SAS Institute Inc., Cary, NC).

## Results

In fiscal years 1999/2000 through 2014/2015 inclusive, we identified 1780 pwRMS in Manitoba, of whom 1,510 had at least one DMT prescription during that time period (Table 1). The DMT-treated and DMT-naïve cohorts had similar sex distribution, year of diagnosis, income and comorbidity distributions. The average age at MS onset was nearly 10 years higher on average in the DMT-naïve group. Forty-five percent of the DMT-treated cohort and 22% of the DMT-naïve cohort had treated relapses. Three-quarters of DMT-treated pwRMS had a MPR >80%. Approximately two-thirds of individuals never switched DMTs, one-quarter switched DMTs once with only one in ten made two or more switches.

**Table 1:** Characteristics of study population.

		Whole cohort *n* (%)	Any DMT use^a^ *n* (%)	DMT naive *n* (%)
Sex	Female	1,332 (74.83)	1,134 (75.1)	198 (73.33)
	Male	448 (25.17)	376 (24.9)	72 (26.67)
Age at MS Onset		39.8 (11)	38.4 (10.4)	47.6 (11.2)
Year of diagnosis	1999/00–2002/03	1,006 (56.52)	865 (57.28)	141 (52.22)
	2003/04–2008/09	442 (24.83)	370 (24.5)	72 (26.67)
	2009/10–2014/15	332 (18.65)	275 (18.21)	57 (21.11)
Income quintiles	1–2	978 (54.94)	829 (54.9)	149 (55.19)
	3–5	802 (45.06)	681 (45.1)	121 (44.81)
Number of comorbidities^b^	0	1,381 (77.58)	1,171 (77.55)	210 (77.78)
	1	331 (18.6)	287 (19.01)	44 (16.3)
	≥2	68 (3.82)	52 (3.44)	16 (5.93)
Number of treated relapses^c^	0	1,046 (58.76)	836 (55.36)	210 (77.78)
	1	366 (20.56)	329 (21.79)	37 (13.7)
	2	150 (8.43)	136 (9.01)	14 (5.19)
	3	76 (4.27)	73 (4.83)	3 (1.11)
	≥4	142 (7.98)	136 (9.01)	6 (2.22)
Adherence^d^	MPR < 80		366 (24.24)	
	MPR ≥ 80		1,144 (75.76)	
Number of DMT switches	0		960 (63.58)	
	1		394 (26.09)	
	2		126 (8.34)	
	3		21 (1.39)	
	4		9 (0.6)	

^a^Use of any DMT at any time over the study period 1999–2015.

^b^Defined using modified Charlson index (10).

^c^Treated relapses defined as the use of high dose oral corticosteroids (>500 mg/day for 3–11 days) or emergency room visits or hospitalizations for MS as most responsible diagnosis (8).

^d^defined by medication possession ratio (MPR).

In the entire cohort of all pwRMS (Table 2), treating DMT exposure as a single-time dependent variable that did not account for treatment adherence or periods on/off treatment, DMT exposure was not associated with time to treated relapse. Male sex was associated with a modestly higher hazard of treated relapse. Conversely, MS diagnosis at a younger age, diagnosis between 2003/04 and 2008/09 fiscal years and a higher household SES were all associated with modestly lower hazard of relapse (Table 2). Comorbidity was not associated with time to first relapse.

**Table 2:** Time to first relapse in all subjects.

Variable		Hazard ratio (95% CIs)
DMT use^**^		1.165 (0.989, 1.372)
Male Sex		1.232 (1.045, 1.452)^a^
Age at diagnosis		0.988 (0.981, 0.995)^a^
Year of diagnosis	2003/04–2008/09	0.688 (0.569, 0.833)^a^
	2009/10–2014/15	0.843 (0.665, 1.069)
Higher income quintiles		0.856 (0.739, 0.992)^a^
Number of Comorbidities	1	1.125 (0.927, 1.364)
	≥2	1.093 (0.722, 1.655)

Cox proportional hazards regression model using DMT exposure as a time-dependent covariate and adjusted for age at diagnosis, sex (female as reference), socioeconomic status (low as reference group) and number of Charlson comorbidities (0 as reference).

^a^*p* < 0.05.

**Figure 1:**
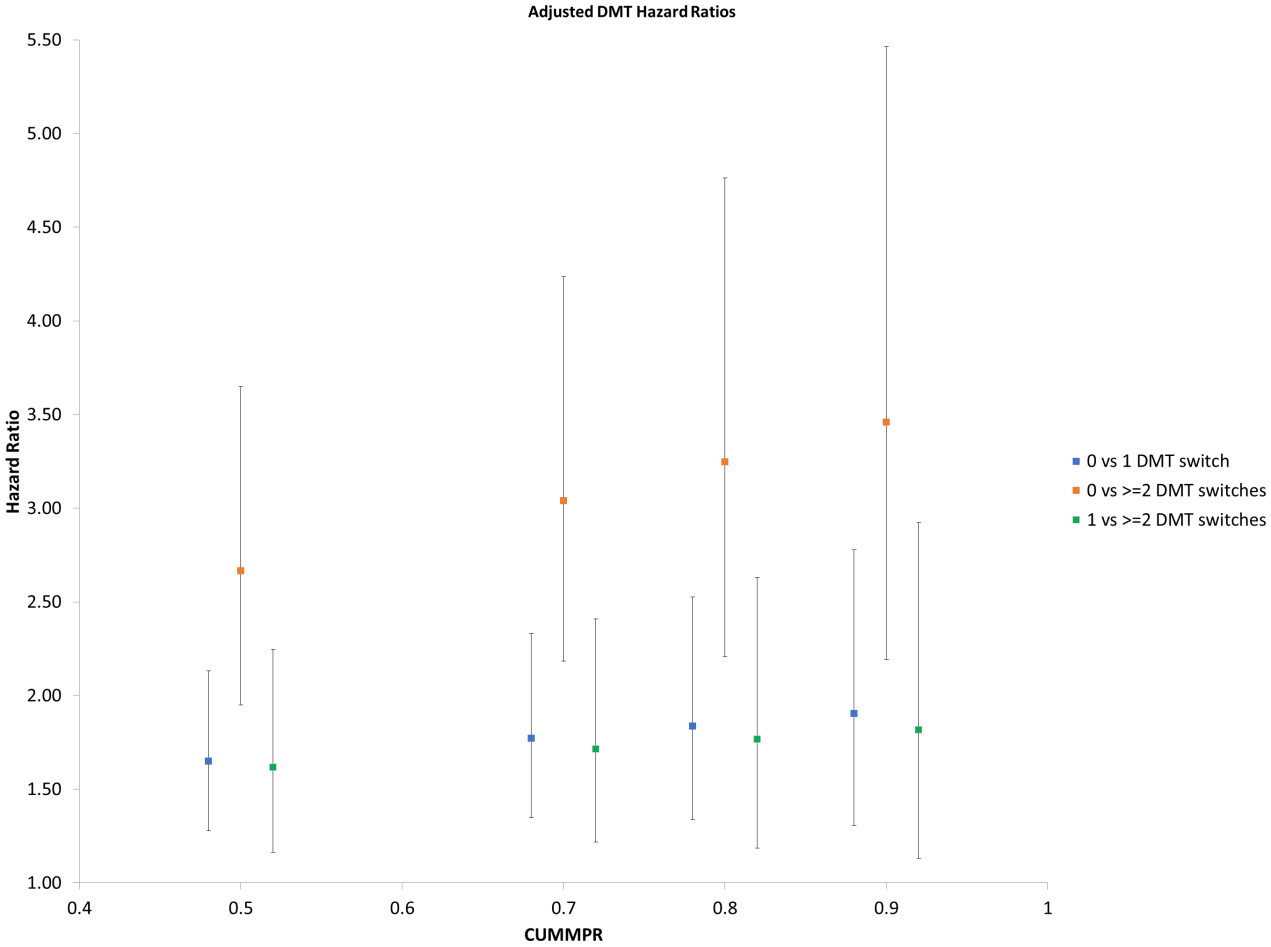
Hazard Ratio of subsequent relapse by number of DMT switches Hazard ratios for subsequent relapse at prespecified CUMMPR thresholds (50, 70, 80, and 90%), comparing individuals who never switched to those who switched once (0 versus 1 switch), those who switched twice (1 versus 2 switches) and those who switched twice (0 versus 2 switches). Age at diagnosis, sex, socioecomonic status, number of comorbidities and year of diagnosis were included as covariates. CUMMPR = cumulative medication possession ratio.

Focusing on pwMS who started DMT, individuals who remained on the same therapy had a lower risk of later relapse at all prespecified CUMMPR thresholds (50, 70, 80 and 90%) than those who switched DMTs (Figure 1). The risk of relapse was similar when comparing individuals who never switched to those who switched once (0 versus 1 switch) and those who switched once to those who switched twice (1 versus 2 switches), but markedly higher when directly comparing those who never switched to those who switched twice (0 versus 2 switches). Age at diagnosis, sex, SES, and number of comorbidities were not associated with time to first-treated serious relapse. Those diagnosed earlier in the study period (2003/04–2008/09) had a lower rate of relapse (HR 0.76; 95%CI: 0.63, 0.93, *p* = 0.008).

Focusing the analysis on direct comparisons between first- and second-line DMTs demonstrated a significantly greater protective effect of second-line therapies (HR 0.44; 95%CI: 0.32–0.62, *p* < 0.0001) in comparison to those who either remained on their initial first-line DMT or switched between first-line DMTs.

## Discussion

In this population-based cohort study, we examined the association between DMT use, including changes in therapy, and time to treated relapse. Adherence to DMT was high in our study population, with three-quarters of individuals having a MPR >80%, comparable to what has been reported in other population-based studies of Canadian MS patients (12–14). Our results demonstrate that switching therapies is associated with a generally higher risk of treated relapse regardless of adherence. This is consistent with treatment switches occurring due to inadequate treatment response. However, we have also shown a robust impact of escalating therapy, with switches to second-line DMTs reducing the hazard of later relapse by approximately 50% compared with those who remained on or switched between first-line medications. This provides a strong rationale for escalating therapy among those who have relapses requiring intervention.

These findings are in keeping with what other groups have shown using different study methodologies. Some groups have used U.S. medical insurance databases to study the impact of DMTs on relapse rates and health care utilization (15–18). One study suggested that initiation of fingolimod resulted in fewer subsequent relapses relative to interferon-β or glatiramer (15) with the others demonstrating that better adherence to DMTs in general was associated with fewer relapses (16–18). Similarly, studies of the multi-national MSBase registry have suggested that switching between first-line injectable DMTs is associated with a higher risk of subsequent relapse in comparison to switching to either fingolimod (19) or natalizumab (20). Our study has the advantage of being done in a single geographical region, with a common set of rules used for treatment switches, including switching to second-line DMTs. Importantly, as provincial regulations require evidence of disease activity on a “first-line” DMT to escalate therapy, including disabling or partially disabling relapses, our results indicates that individuals who generally had more active disease still had better relapse outcomes after switching to second-line agents.

Although the focus of our study was on the association between DMT switches and relapses, other outcomes are important from patient, clinician and health system perspectives. A recent study using data from four Canadian provinces, including Manitoba, found that exposure to any DMT was associated with a survival advantage (21). In British Columbia, Canada, use of any DMT was associated with reduced hospitalization rates among individuals under age 55 years, but not with rates of physician visits (22). An Australian study reported that use of higher efficacy therapy was associated with improved self-reported work outcomes (23). However, the specific effect of switching strategies for DMT on survival, health care use and employment were not examined, and warrant future study.

Our study has some limitations. As we are not able to capture use of intravenous steroids in the administrative databases, we are underestimating the number of treated relapses before 2009 when the use of high-dose oral steroids became the standard of care in our province (9). This likely accounts for the higher relapse rate in the later years of our study. However, that period largely predates the availability of second-line DMTs, as the first Health Canada-approved second-line DMT (natalizumab) was not covered on the Manitoba provincial formulary until 2010, so this does not have a significant impact on our demonstration that second-line DMTs were associated with lower relapse rates. Moreover, this would not affect between group comparisons. Prior studies have shown that females have higher overall relapse rates. The higher relapse rate we observed in males might reflect the previously documented higher rates of severe relapses in males (24) as our algorithm only captures treated relapses.

While MPR demonstrates how often prescriptions are being filled, from administrative data alone we cannot directly measure whether the DMT was taken correctly and consistently. From administration data, it is not possible to identify all potential reasons, such as side effects, for medication switches nor can some potential confounders, such as smoking status be captured. Given the relatively small numbers of patients switching, we could not separate specific-DMT treatment effects. The modeling used did not account for potential changes in SES or comorbidity status over time.

Our analysis of DMT use in an entire Canadian province has the advantage of being a geographical based cohort, across a region with a single health care system and uniform access to MS specialist care, and consistent policies regarding access to DMT, including second-line therapies. We used a validated case definition which we have previously shown to accurately identify serious MS relapses requiring therapy (9). Importantly, we have also shown the advantage of using a more nuanced modeling approach with multiple covariates to account for different aspects of DMT use and exposure to better capture how medications are used in real-world settings. Incorporating covariates to measure adherence, treatment breaks and switches between different DMTs showed a beneficial effect of DMT use on the time-to-first relapse which was not seen when DMT use was treated as a single time-dependent covariate in a model of all pwRMS. For analyses comparing the effects of switches between first-line DMTs versus switches from first-line and second-line DMTs we opted to use regression-based analyses rather than propensity scores as traditional regression-based methods perform as well as propensity-based methods, particularly when there are more than 8–10 events per covariate (25, 26). These methods could be used in further population-based studies of newer DMTs.

Escalation of DMT in pwRMS is effective in reducing the rate of relapses among individuals with a history of treated relapses. Ongoing pragmatic clinical trials will address whether initial treatment with higher efficacy DMTs rather than an escalation approach improves outcomes in treatment-naïve individuals with a range of disease activity (27, 28).

## Data availability statement

The data analyzed in this study was obtained from the Manitoba Centre for Health Policy (MCHP) Population Health Research Data Repository under project 2015-022 (HIPC 2015/2016-05), the following licenses/restrictions apply: the data used in this study were derived from administrative health and social data as a secondary use. The data were provided under specific data sharing agreements only for approved use at MCHP. Where necessary, source data specific to this article or project may be reviewed at MCHP with the consent of the original data providers, along with the required privacy and ethical review bodies. Requests to access these datasets should be directed to MCHP, info@cpe.umanitoba.ca.

## Ethics statement

The studies involving humans were approved by the University of Manitoba Health Research Ethics Board. The studies were conducted in accordance with the local legislation and institutional requirements. The participants provided their written informed consent to participate in this study.

## Author contributions

JM: conceptualization, methodology, project administration, funding acquisition, and writing of original draft, review, and editing. OE: conceptualization, methodology, writing—review, and editing. RF: conceptualization, funding acquisition, writing—review, and editing. RM: conceptualization, methodology, funding acquisition, writing—review, and editing. All authors contributed to the article and approved the submitted version.

## Funding

This work was supported by a grant from the Multiple Sclerosis Society of Canada (2275).

## Conflict of interest

JM has received research support from the Research Manitoba, Multiple Sclerosis Scientific Research Foundation, Consortium of MS Centers and Manitoba Medical Service Foundation and for MS trials from Biogen Idec, Roche, SanofiAventis and honoraria from Biogen Idec, Roche, and EMD Serono. OE and RF has nothing to disclose. RM has received research funding from the Canadian Institutes of Health Research, Research Manitoba, Multiple Sclerosis Society of Canada, Multiple Sclerosis Scientific Research Foundation, Crohn’s and Colitis Canada, US Department of Defense, CMSC, and National Multiple Sclerosis Society. She is a co-investigator on studies funded by the Biogen Idec and Roche. She serves on the Editorial Boards of Neurology and Multiple Sclerosis Journal.

## Publisher’s note

All claims expressed in this article are solely those of the authors and do not necessarily represent those of their affiliated organizations, or those of the publisher, the editors and the reviewers. Any product that may be evaluated in this article, or claim that may be made by its manufacturer, is not guaranteed or endorsed by the publisher.
