# Supplementary material for: Switching to second line MS disease-modifying therapies is associated with decreased relapse rate
Source: Front Neurol. 2023 Sep 6;14:1243589. doi: 10.3389/fneur.2023.1243589 (PMC10511745; doi:10.3389/fneur.2023.1243589)
Supplement: Supplementary file 1 [file Data_Sheet_1.PDF]

Supplemental Table: International Classification of Disease (ICD)-9/10 claim code for any central nervous system demyelinating disease		
	**ICD-9-CM**	**ICD-10-CA**
optic neuritis	377.3	H46
acute transverse myelitis	323.82	G37
acute disseminated encephalomyelitis	323	G36.9
demyelinating disease of CNS unspecified	341.9	G37.8
other acute disseminated demyelination		G36
MS	340	G35
neuromyelitis optica	341.0	G36.0
